# *De Novo* Assembly and Transcriptome Analysis of Wheat with Male Sterility Induced by the Chemical Hybridizing Agent SQ-1

**DOI:** 10.1371/journal.pone.0123556

**Published:** 2015-04-21

**Authors:** Qidi Zhu, Yulong Song, Gaisheng Zhang, Lan Ju, Jiao Zhang, Yongang Yu, Na Niu, Junwei Wang, Shoucai Ma

**Affiliations:** National Yangling Agricultural Biotechnology & Breeding Center, Yangling Branch of State Wheat Improvement Centre, Wheat Breeding Engineering Research Center, Ministry of Education, Key Laboratory of Crop Heterosis of Shaanxi Province, Northwest A&F University, Yangling, Shaanxi, China; Nanjing Agricultural University, CHINA

## Abstract

Wheat (*Triticum aestivum* L.), one of the world’s most important food crops, is a strictly autogamous (self-pollinating) species with exclusively perfect flowers. Male sterility induced by chemical hybridizing agents has increasingly attracted attention as a tool for hybrid seed production in wheat; however, the molecular mechanisms of male sterility induced by the agent SQ-1 remain poorly understood due to limited whole transcriptome data. Therefore, a comparative analysis of wheat anther transcriptomes for male fertile wheat and SQ-1–induced male sterile wheat was carried out using next-generation sequencing technology. In all, 42,634,123 sequence reads were generated and were assembled into 82,356 high-quality unigenes with an average length of 724 bp. Of these, 1,088 unigenes were significantly differentially expressed in the fertile and sterile wheat anthers, including 643 up-regulated unigenes and 445 down-regulated unigenes. The differentially expressed unigenes with functional annotations were mapped onto 60 pathways using the Kyoto Encyclopedia of Genes and Genomes database. They were mainly involved in coding for the components of ribosomes, photosynthesis, respiration, purine and pyrimidine metabolism, amino acid metabolism, glutathione metabolism, RNA transport and signal transduction, reactive oxygen species metabolism, mRNA surveillance pathways, protein processing in the endoplasmic reticulum, protein export, and ubiquitin-mediated proteolysis. This study is the first to provide a systematic overview comparing wheat anther transcriptomes of male fertile wheat with those of SQ-1–induced male sterile wheat and is a valuable source of data for future research in SQ-1–induced wheat male sterility.

## Introduction

Wheat (*Triticum aestivum* L.) is one of the most important cultivated crops in the global food system; it supplies the needed food protein and calories for over 35% of the world’s population and is the principal food in more than 40 countries [1–4]. About 700 million tons are produced annually, and so far, production has kept abreast of world population growth. By 2025, wheat production must increase to 1,200 million tons in order to meet the needs of the growing world population. In other words, wheat grain production must increase at an average annual rate of about 2% in the limited land area that is cultivated [5–7]. The most efficient way to increase wheat productivity is to use wheat heterosis to improve yield and growing characteristics [8].

Heterosis (or hybrid vigor) is a universal phenomenon in biology, and hybrid offspring often display increased yield, higher total biomass, and improved abiotic and biotic stress resistance compared with their homozygous parents [9–12]. Hybrid breeding has been a brilliant success in many allogamous crop species, including corn, sunflower, brassicas, cucurbits, carrots, beets, and onions, and it also plays a major role in some autogamous cereals [13–15]. For instance, about 80% of the oilseed rape in China is a hybrid cultivar, and hybrid sorghum dominates the U.S. and world markets [16]. Since the late 1970s, the area of agricultural land used for hybrid rice cultivation has rapidly increased throughout Asia, particularly in China, India, and Vietnam. More than 50% of the total rice-growing area is planted with hybrid rice cultivars, which have fairly high yields compared with conventional varieties [17–19]. Due to the small size and close proximity of male and female reproductive organs in wheat, it is difficult to perform hand emasculation in hybrid wheat breeding. Chemical hybridization agents are good tools that can produce hybrid wheat, inducing wheat physiological male sterility (PMS) without harming the pistil [20–22].

SQ-1, an ideal chemical hybridization agent, is a systemic product that is efficiently transported in wheat from leaves to flowers [5]. It is effective and safe, has low phytotoxic effects, allows flexibility in the application stage, most importantly, allows many thousands of hybridized combinations to be combined freely [23–26]. SQ-1 is widely applied in hybrid wheat production; however, the mechanism by which SQ-1 induces male sterility in wheat remains unclear. In PMS, the anther tapetum degenerates earlier, at the mononuclear stage; the majority of microspores fail to divide; and only a few microspores develop to achieve bicellular status [27, 28]. The inducement of wheat PMS is a complicated metabolic process that affects the plant’s defense system against oxidation stress, signal transduction and transcriptional regulation, energy and protein metabolism, reactive oxygen species (ROS) metabolism, and the ubiquitin-26S proteasome pathway [29–33].

In recent years, next-generation sequencing (NGS) techniques (such as Roche 454, Illumina Solexa GA, and ABI SOLID) have rapidly developed and have provided efficient, powerful, and inexpensive tools for advanced research in many fields, including genome re-sequencing, small RNA sequencing, Degradome sequencing, and *de novo* transcriptome sequencing for non-model organisms [34–38]. RNA sequencing (RNA-Seq) has been applied as a tool for transcriptome analysis in many species, such as *Arabidopsis thaliana* [39], *Brassica rapa* [40], chili pepper [41], sweet orange [42], radish [43], rice [44], and corn [45]. As pollen development is known to be regulated by transcription levels, whole-transcriptome analysis was utilized to explore unigenes that were involved in anther development [46].

The aim of this study was to seek out the key genes and important metabolic pathways associated with wheat PMS. It is the first study to characterize the complete transcriptome of wheat PMS using high-throughput Illumina HiSeq 2500 sequencing technology, and it provides new insights into wheat male sterility induced by SQ-1.

## Materials and Methods

### Chemical hybridizing agent SQ-1

SQ-1 was obtained from the Key Laboratory of Crop Heterosis of Shaanxi Province and used to produce hybrid wheat seed. It is a pyridazinecarboxylic acid compound that is readily soluble in water. Stamen sterility (male sterility rate > 98%) was induced without affecting pistil fertility when 5.0 kg ha^-1^ SQ-1 was evenly sprayed on the leaves of wheat at the 8.5 stage of the Feekes scale.

### Plant material and sample preparation

The wheat cultivar Xi-nong 1376 was sown in the experimental field of Northwest A & F University, Yangling, Shaanxi, China (108°E, 34°15′N) on October 7, 2013. The experimental plot contained about 9,000 plants grown in 180 rows (1.5 m long each) at a density of 25 cm space between rows and 3 cm between plants with a row, it was divided into two groups (treatment group and control group) and each group contained 90 rows. When the average growth stage of the wheat reached the 8.5 stage of the Feekes scale, 5.0 kg ha^-1^ SQ-1 was evenly sprayed on the leaves of the wheat in the treatment group, and the same amount of water was sprayed on the leaves of the wheat in the control group. At the uninucleate pollen stage of wheat, anthers were separately collected from wheat fertility spikes and SQ-1–induced male sterile spikes, frozen immediately in liquid nitrogen, and stored at -80°C until use.

### RNA extraction and preparation of library for Illumina sequencing

Each frozen anther was ground in a mortar with liquid nitrogen, after which total RNA was extracted using Trizol Reagent (Invitrogen Life Technologies, USA) according to the standard protocol. The quality of total RNA was checked by electrophoresis in a 1.5% agarose gel and the concentration of total RNA was determined by NanoDrop (Thermo Scientific, Wilmington, DE, USA). The RNA integrity value was further verified using an Agilent 2100 Bioanalyzer (Agilent Technologies, Santa Clara, CA, USA). The two cDNA libraries of fertile wheat and SQ-1–induced male sterile wheat were prepared according to the manufacturer’s instructions for mRNA-Seq sample preparation (Illumina, Inc., San Diego, CA, USA). The cDNA library products were sequenced by Illumina paired-end sequencing technology with read lengths of 100 bp, and they were sequenced on the Illumina HiSeq 2500 instrument by Biomarker Technologies Co. Ltd. (Beijing, China). The dataset was submitted to the NIH Short Read Archive (accession number: SRP051670).

### Sequence data analysis and *de novo* assembly

Before assembly, the raw paired-end reads were filtered to obtain high-quality clean reads. Low-quality sequences were removed, including sequences with ambiguous bases (denoted with an “N” in the sequence trace) and reads with more than 20% low-quality bases (quality value ˂ 20). After purity filtering was completed, the high-quality reads were assembled by Trinity (release 20131110) with default parameters to construct unique consensus sequences [47].

### Analysis of differential gene expression

The gene expression level was measured by the values of RPKM (reads per kb per million reads) [48]. Unigenes that were differentially expressed between the male fertile and SQ-1–induced male sterile wheat were analyzed by Chi-square test using IDEG6 software (http://telethon.bio.unipd.it/bioinfo/IDEG6/). The false discovery rate (FDR) method was introduced to determine the threshold *p*-value at FDR ˂ 0.01, and the absolute value of log_2_Ratio ≥ 1 was used as the threshold to determine the significance of the differential expression of unigenes.

### Gene annotation and classification

In order to perform functional annotation, the assembled unigenes were submitted to public databases and compared with the NCBI non-redundant protein database (NR) [49], the Swiss-Prot database (http://www.uniprot.org/) [50], the Clusters of Orthologous Groups (COG) database (http://www.ncbi.nlm.nih.gov/COG/) [51], and the Kyoto Encyclopedia of Genes and Genomes (KEGG) database (http://www.genome.jp/kegg/) [52] using BlastX (v. 2.2.26) with an E-value of less than 1e-5, while the Gene Ontology (GO) annotations were analyzed using the Blast2GO (v. 2.5) program (http://www.geneontology.org/) [53]. All differentially abundant unigenes between the male fertile and SQ-1–induced male sterile wheat were mapped to the GO and KEGG pathway databases, and then the respective numbers of unigenes for every GO term and KEGG Orthology (KO) term were calculated. To compare these unigenes with the whole transcriptome background for wheat, significantly enriched GO and KO terms from the set of differentially abundant unigenes were identified using the hypergeometric test. The formula for the gene enrichment test was

$$P=1-\sum_{i=0}^{m-1}\frac{\left(\begin{array}{l} M\\ i \end{array}\right)\left(\begin{array}{l} N-M\\ n-i \end{array}\right)}{\left(\begin{array}{l} N\\ n \end{array}\right)}$$

in which *N* represents the total number of unigenes with GO and KEGG pathway annotation; *n* represents the number of differentially abundant unigenes in *N*; *M* represents the number of unigenes that were annotated to certain GO or KO terms; and *m* represents the number of differentially abundant unigenes in *M*. The initial *p*-values were then adjusted using a Bonferroni Correction and a corrected *p*-value of 0.05 was adopted as a threshold.

## Results

### Illumina sequencing and *de novo* assembly

**Fig 1:**
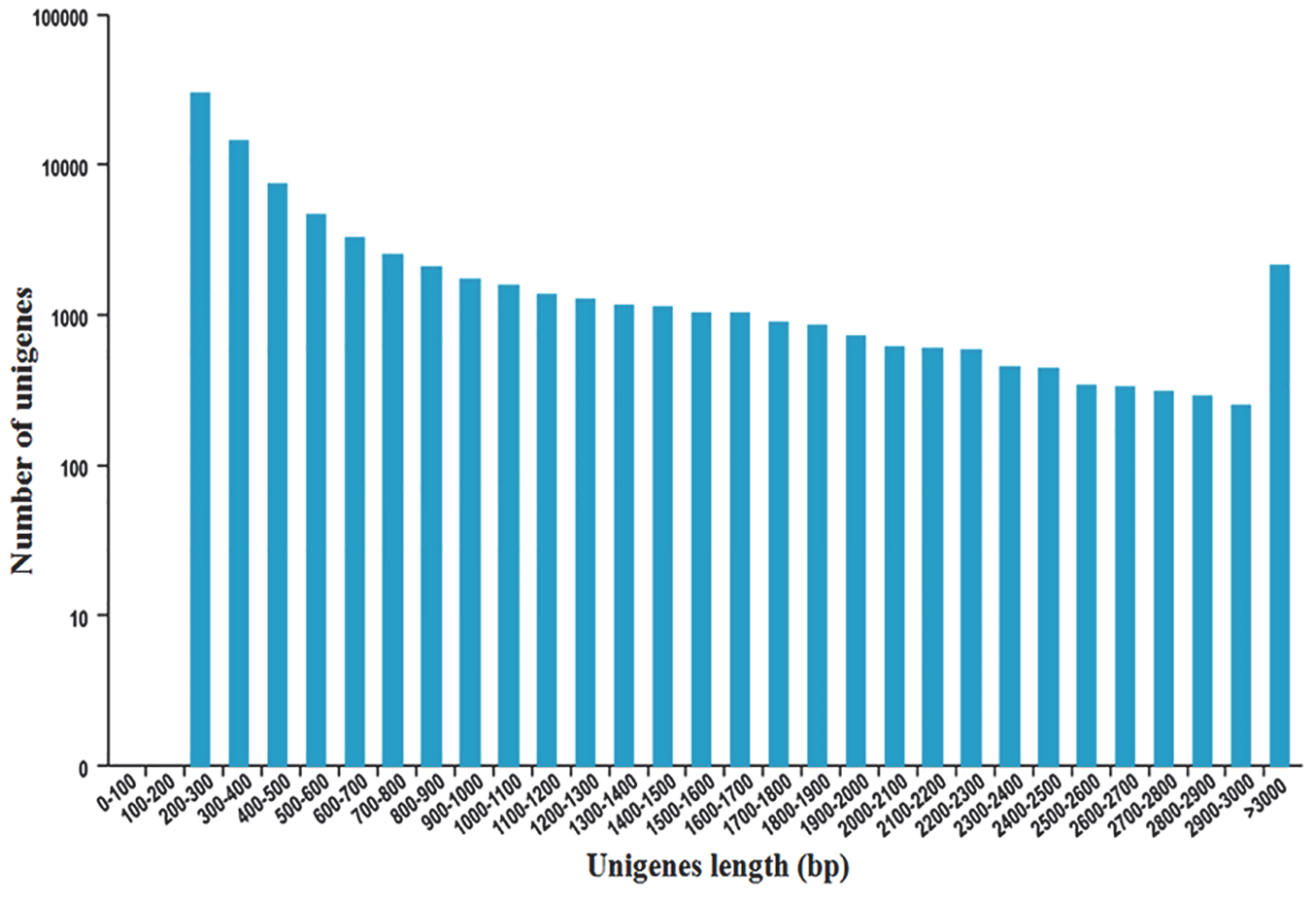
Distribution of lengths of the assembled unigenes in wheat anthers.

A total of 42,634,123 paired-end sequence reads (20,586,618 and 22,047,505 for fertile wheat and SQ-1–induced male sterile wheat, respectively) remained, with the Q30 percentage over 92%. The high-quality reads were aligned to assembled unigenes, with more than 80% of the high-quality reads mapping to a unique or to multiple unigene locations (Table 1). Trinity (release 20131110) software was used to generate 4,062,157 contigs, which were further assembled into 82,356 unigenes with an average length of 724 bp and an N50 length of 1,289 bp. The majority of the unigenes (61.93%) were 200–500 bp long. There were 25,042 unigenes (30.41%) in the 500–2000 bp range and 6,309 unigenes (7.66%) that exceeded 2000 bp (Fig 1).

**Table 1:** Summary of transcriptome sequencing data in the fertile wheat and SQ-1–induced male sterile wheat.

	Fertile wheat	Male sterile wheat
Read length (bp)	100 + 100	100 + 100
Total bases	4,158,157,525	4,453,231,425
Total reads	20,586,618	22,047,505
Total mapping reads	16,543,508 (80.36%)	17,712,696 (80.34%)
Uniquely-mapping reads	6,169,183 (37.29%)	6,772,690 (38.24%)
Multiply-mapping reads	10,374,325 (62.71%)	10,940,006 (61.76%)
GC (%)	51.39	52.44
Q30^a^ (%)	92.87	92.63

^a^ indicates the percentage of sequences at a sequencing error rate of less than 0.1%

### Prediction of open reading frames

We used GetORF software from the EMBOSS (v. 6.0.1) analysis package to perform open reading frame (ORF) predictions. A total of 81,838 ORFs were detected from 82,356 unigenes, with an average length of 402 bp and an N50 length of 846 bp. The majority of ORFs (68.31%) contained 0–300 bp, whereas 17,566 ORFs (21.46%) contained 300–1000 bp and 8,369 ORFs (10.23%) exceeded 1,000 bp (Table 2). The remaining 518 unigenes with no ORFs were non-coding sequences or likely originated from untranslated regions.

**Table 2:** Statistics for open reading frame (ORF) predictions.

	Total ORFs	Percentage (%)
0–300 bp	55,903	68.31
300–500 bp	9,469	11.57
500–1000 bp	8,097	9.89
1000–2000 bp	6,232	7.62
2000–3000 bp	1,494	1.83
>3000 bp	643	0.79
Total number	81,838	-
Total length (bp)	32,904,006	-
N50 length (bp)	846	-
Mean length (bp)	402	-

### Analysis of differentially expressed unigenes

A sequence similarity search was conducted against several public databases: the NR database, the Swiss-Prot database, the COG database, the GO database, and the KEGG database, using Blast with an E-value of less than 1e-5. A total of 967 differentially expressed unigenes (88.88%) exhibited gene annotation (Table 3).

**Table 3:** Summary statistics of functional annotation for differentially expressed unigenes.

Annotated databases	Differentially expressed unigenes	Percentage (%)
COG^b^	333	30.61
GO^c^	637	58.55
KEGG^d^	156	14.34
NR^e^	967	88.88
Swiss-Prot	756	69.49
Total	967	88.88

^b^ Clusters of Orthologous Groups database

^c^ Gene Ontology database

^d^ Kyoto Encyclopedia of Genes and Genomes database

^e^ NCBI non-redundant protein database

**Fig 2:**
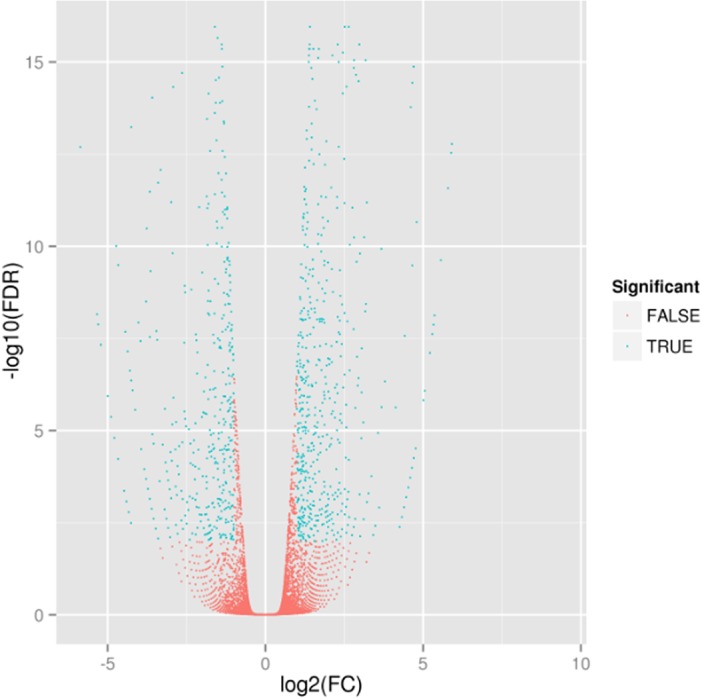
Changes in the abundance of unigenes between fertile wheat and SQ-1–induced male sterile wheat. The further log_2_(FC) deviates from 0, the greater the difference in fertile wheat and SQ-1–induced male sterile wheat. Higher values of FDR indicate more reliable results of unigenetic differential expression.

**Fig 3:**
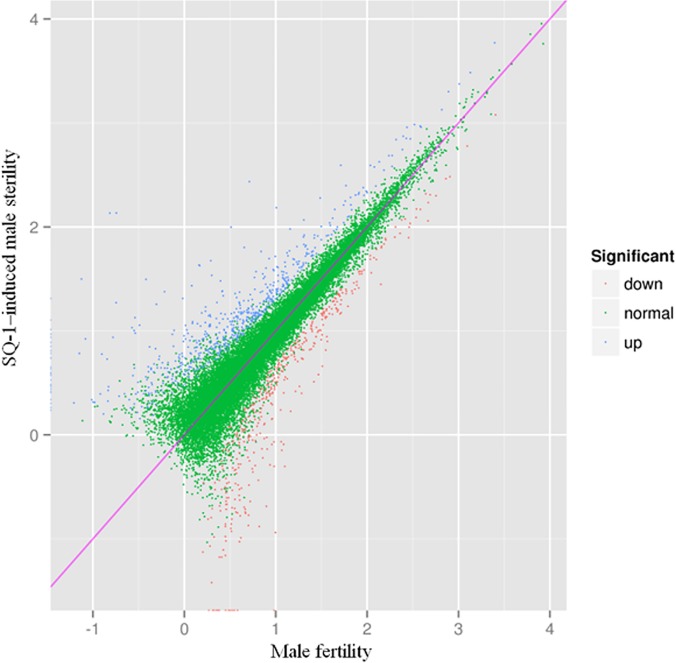
Differentially expressed genes between fertile wheat and SQ-1–induced male sterile wheat. A correlation plot of 643 up-regulated unigenes (blue) and 445 down-regulated unigenes (red) in SQ-1–induced male sterile wheat.

To identify differentially expressed unigenes between fertile wheat and SQ-1–induced male sterile wheat, putative differentially expressed unigenes were identified on the basis of RPKM values that were calculated from the read counts mapped onto the reference transcriptome. A total of 1,088 unigenes were differentially expressed between fertile wheat and SQ-1–induced male sterile wheat according to a comparison of expression levels with Fold Change (FC) ≥ 2 and FDR ˂ 0.01 (Fig 2). Using the fertile wheat as a reference, 643 up-regulated unigenes (with higher levels of expressions in the SQ-1–induced male sterile wheat) and 445 down-regulated unigenes (with higher levels of expressions in the fertile wheat) were identified (Fig 3). Significantly more unigenes were up-regulated in sterile wheat than were down-regulated. The unigenes with the two highest levels of up-regulation were involved in “posttranslational modification, protein turnover, chaperones” and “oxidation-reduction process,” while the two highest levels of down-regulation corresponded to “translation, ribosomal structure and biogenesis” and “amino acid transport and metabolism.”

### GO functional classification of differentially expressed unigenes

**Fig 4:**
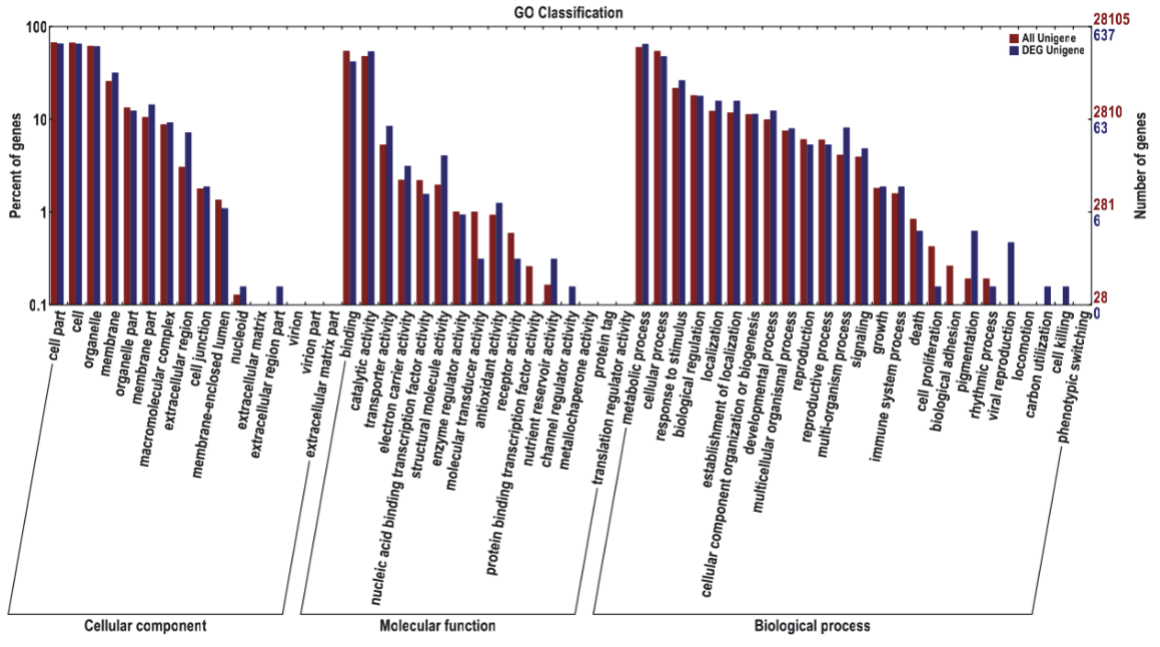
Gene Ontology (GO) classifications of assembled unigenes. A total of 637 differentially expressed unigenes between fertile wheat and SQ-1–induced male sterile wheat were assigned to 57 subcategories using GO assignment (blue), and all assembled unigenes were assigned to 57 subcategories (red).

GO is an international classification system for standardized gene functions that may be obtained from the NR annotation information. The GO terms consist of the following three broad categories: cellular components, molecular functions, and biological processes. GO assignment was used to assign 637 differentially expressed unigenes to 57 subcategories. Among them, 394 differentially expressed unigenes were related to cellular components, 487 differentially expressed unigenes were grouped under molecular functions, and 492 differentially expressed unigenes were involved in biological processes. Within the cellular component category, the majority of differentially expressed unigenes were enriched in the subcategories of “cell part,” “cell,” and “organelle.” Within the biological process category, the great majority were related to “metabolic process,” “cellular process,” and “response to stimulus.” Within the molecular function category, the largest proportion of differentially expressed unigenes were involved in “binding” and “catalytic activity,” and a relatively large number were related to “transporter activity” (Fig 4), which may play an important role in ions, small molecules, and macromolecules transport, such as amino acids and proteins.

### COG functional classification of differentially expressed unigenes

**Fig 5:**
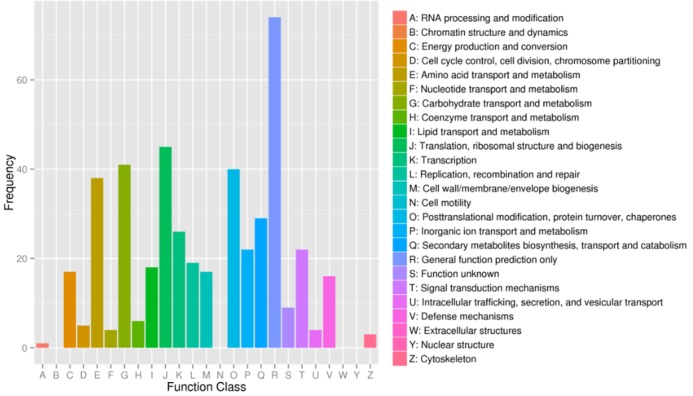
Clusters of Orthologous Groups (COG) functional classifications of the differentially expressed unigenes in wheat.

All the differentially expressed unigenes were aligned to a search against the COG database for functional prediction and classification, and 333 unigenes were assigned to 25 COG functional categories. Among these categories, the cluster of “general function prediction only” was the largest group (22.22%), followed by “translation, ribosomal structure and biogenesis” (13.51%), “carbohydrate transport and metabolism” (12.31%), “posttranslational modification, protein turnover and chaperones” (12.01%), and “amino acid transport and metabolism” (11.41%). The smallest groups were “nucleotide transport and metabolism” (1.20%), “cytoskeleton” (0.90%), and “RNA processing and modification” (0.30%) (Fig 5).

### KEGG pathway mapping of differentially expressed unigenes

Functional classification and pathway assignment were performed using BlastX with an E-value of less than 1e-5. A total of 156 differentially expressed unigenes were annotated, and 126 were mapped onto 60 pathways in the KEGG database. The pathways containing the most differentially expressed unigenes were involved in “ribosome” (20 unigenes), followed by “photosynthesis” (8 unigenes), “fatty acid biosynthesis” (7 unigenes), “nitrogen metabolism” (7 unigenes), “arginine and proline metabolism” (6 unigenes), “biosynthesis of unsaturated fatty acids” (6 unigenes), “carbon fixation in photosynthetic organisms” (6 unigenes), “glutathione metabolism” (6 unigenes), “glycolysis/gluconeogenesis” (6 unigenes), “RNA transport” (6 unigenes), “starch and sucrose metabolism” (5 unigenes), “galactose metabolism” (4 unigenes), “peroxisome” (4 unigenes), “oxidative phosphorylation” (3 unigenes), and “ubiquitin-mediated proteolysis” (3 unigenes) (Table 4).

**Table 4:** Differentially expressed unigenes with significantly enriched pathways.

Pathway	DEG^f^	Total^g^	Enrichment factor	*P*-value
Ribosome	20	331	0.18	0.05
Photosynthesis	8	86	0.47	0.16
Fatty acid biosynthesis	7	46	0.25	0.02
Nitrogen metabolism	7	62	0.3	0.10
Glycolysis / Gluconeogenesis	6	186	0.26	1
Arginine and proline metabolism	6	91	0.29	1
Glutathione metabolism	6	89	0.42	1
Carbon fixation in photosynthetic organisms	6	125	0.43	1
Biosynthesis of unsaturated fatty acids	6	56	0.41	0.27
RNA transport	6	284	0.32	1
Alanine, aspartate and glutamate metabolism	5	73	0.08	1
Starch and sucrose metabolism	5	148	0.43	1
Glyoxylate and dicarboxylate metabolism	5	51	0.46	0.81
Plant hormone signal transduction	5	188	0.46	1
Protein processing in endoplasmic reticulum	5	195	0.59	1
Galactose metabolism	4	65	0.17	1
Phenylalanine metabolism	4	65	0.47	1
Protein export	4	61	0.22	1
Peroxisome	4	99	0.54	1
Fructose and mannose metabolism	3	83	0.25	1
Ascorbate and aldarate metabolism	3	50	0.48	1
Oxidative phosphorylation	3	195	0.49	1
Photosynthesis—antenna proteins	3	34	0.52	1
Purine metabolism	3	206	0.64	1
Glycine, serine and threonine metabolism	3	68	0.7	1
Cysteine and methionine metabolism	3	121	0.39	1
Amino sugar and nucleotide sugar metabolism	3	113	0.71	1
Phenylpropanoid biosynthesis	3	76	0.48	1
Basal transcription factors	3	58	0.83	1
Ubiquitin mediated proteolysis	3	119	0.78	1
Plant-pathogen interaction	3	107	0.53	1
Pentose phosphate pathway	2	80	0.87	1
Ubiquinone and other terpenoid-quinone biosynthesis	2	34	0.59	1
Pyrimidine metabolism	2	160	0.59	1
Tyrosine metabolism	2	37	0.77	1
Tryptophan metabolism	2	55	0.79	1
Glycerolipid metabolism	2	56	0.67	1
Sulfur metabolism	2	35	0.79	1
mRNA surveillance pathway	2	180	1	1
Citrate cycle (TCA cycle)	1	73	0.9	1
Fatty acid metabolism	1	63	0.9	1
Valine, leucine and isoleucine biosynthesis	1	61	1.06	1
Benzoxazinoid biosynthesis	1	3	1.06	1
Cyanoamino acid metabolism	1	24	1.1	1
Glycerophospholipid metabolism	1	92	1.11	1
Sphingolipid metabolism	1	19	1.12	1
Glycosphingolipid biosynthesis—globo series	1	9	1.13	1
Pyruvate metabolism	1	108	1.18	1
Butanoate metabolism	1	42	1.55	1
Pantothenate and CoA biosynthesis	1	28	1.33	1
Limonene and pinene degradation	1	21	1.71	1
Carotenoid biosynthesis	1	32	1.77	1
Zeatin biosynthesis	1	17	2.05	1
Flavonoid biosynthesis	1	32	2.22	1
Flavone and flavonol biosynthesis	1	8	1.83	1
Stilbenoid, diarylheptanoid and gingerol biosynthesis	1	21	2.59	1
Glucosinolate biosynthesis	1	6	1.93	1
RNA polymerase	1	79	2.25	1
DNA replication	1	55	3.04	1
Circadian rhythm—mammal	1	14	2.53	1

^f^ the number of differentially expressed unigenes (DEGs)

^g^ the number of all unigenes

In all, 72 down-regulated unigenes were assigned to 40 pathways. The pathways containing the largest number of down-regulated unigenes were involved in “ribosome” (18), a large and complex molecular machine that serves as the primary site of biological protein synthesis. RNA polymerase may be closely involved in fertility, and DNA-directed RNA polymerase II subunit RPB1 was down-regulated in SQ-1–induced male sterile wheat. In addition, 3 down-regulated unigenes were involved in “purine metabolism” and 2 down-regulated unigenes were related to “pyrimidine metabolism.”

We identified 17 down-regulated unigenes related to photosynthesis, including “photosynthetic electron transport chains” (8), “carbon fixation in photosynthetic organisms” (6), and “photosynthesis-antenna proteins” (3). Previous studies showed that SQ-1–induced wheat male sterility was closely linked with plant respiration, and the short supply of energy ultimately led to wheat male sterility [30]. Our results showed that down-regulated unigenes were involved in “glycolysis/gluconeogenesis” (3 unigenes), “citrate cycle” (1 unigene), “pentose phosphate pathway” (2 unigenes), and “oxidative phosphorylation” (2 unigenes).

ROS are chemically reactive molecules containing oxygen, including hydroxyl radical (OH), lipid peroxide (ROO–), singlet oxygen (^1^O_2_), superoxide radical (O_2_^–^), and hydrogen peroxide (H_2_O_2_), which can lead to the significant destruction of cells [54]. Catalase and peroxidase were dramatically down-regulated in SQ-1–induced male sterile wheat. In addition, 3 down-regulated unigenes were related to “plant–pathogen interaction” (pathogenesis-related protein 1, jasmonate ZIM domain-containing protein, and calmodulin) and play important roles in the response to environmental stress. A few down-regulated unigenes were involved in “amino acid metabolism,” including glutamate, tryptophan, glycine, and methionine.

Glutathione is an abundant and important antioxidant in plants that can react with electrophilic or oxidizing species before the latter can damage more critical cellular constituents, such as nucleic acids and proteins [55]. Among all the up-regulated unigenes, the paths containing the largest number of up-regulated unigenes were involved in “glutathione metabolism” (6 unigenes). A few up-regulated unigenes were related to protein degradation, including “protein processing in endoplasmic reticulum” (5 unigenes), “ubiquitin mediated proteolysis” (3 unigenes), and “protein export” (4 unigenes). mRNA degradation plays an important role in gene expression regulation, and 2 up-regulated unigenes were involved in “mRNA surveillance pathway,” which contained the nonsense-mediated mRNA decay and no-go decay. In addition, 2 up-regulated unigenes were closely interrelated with anaerobic respiration, a form of respiration using electron acceptors other than oxygen that is generally less efficient energetically than aerobic respiration.

## Discussion

Wheat PMS is closely related to material metabolism and energy metabolism and is characterized by little starch accumulation in the mature pollen and deformed shape of abortive pollen particles [29]. As a key regulator of pyruvate metabolism, pyruvate dehydrogenase complex (PDC) links the glycolysis metabolic pathway to the citric acid cycle. PDC-*E1α* was down-regulated in PMS wheat relative to fertile wheat, a change that results in an energy shortage during pollen development [32]. Furthermore, some studies showed that the expression level of three phosphoric acid–glycerol dehydrogenase (GAPDH) genes in the PMS lines were lower than those in fertile lines, The reduced transcription of GAPDH genes in wheat anthers were closely associated with pollen abortion [30].

The expression level of the gene for aconitase, a key enzyme in the tricarboxylic acid cycle, was significantly lower in PMS wheat anthers than that in fertile anthers at the binucleate and trinucleate stages. The shortage of energy and the production of some metabolic intermediates caused by abnormal expression of the aconitase gene probably induced pollen abortion [29]. Our results showed that a large number of unigenes related to material and energy metabolism were down-regulated in PMS wheat, including ribulose-bisphosphate carboxylase, glyceraldehyde-3-phosphate dehydrogenase, fructose-bisphosphate aldolase, and pyruvate dehydrogenase. These genes were involved in the categories of “photosynthesis” (8 unigenes), “carbon fixation in photosynthetic organisms” (6 unigenes), “citrate cycle” (1 unigene), “glycolysis/gluconeogenesis” (3 unigenes), and “starch and sucrose metabolism” (2 unigenes).

The amount of ROS can increase sharply under conditions such as ultraviolet light, environmental stress (including cold, heat, salt, drought, and heavy metals stress), or anthropic action through xenobiotics, such as herbicides [54]. Increased ROS metabolism was closely related to pollen abortion in PMS wheat. From a late stage of mononucleate pollen to the initial stage of binonucleate pollen, the rate of generation of O_2_^−^ and the amounts of H_2_O_2_ and MDA of PMS wheat anthers were significantly higher than those of fertile anthers, but the activity levels of SOD, POD, CAT, and APX were significantly lower than those of fertile anthers. The serious imbalance in ROS metabolism and the increase in membrane lipid peroxidation were probably the major cause of pollen abortion [31]. In our study, we found that the activities of POD and CAT were significantly higher in fertile anthers than in PMS wheat anthers, and they affected the normal development of wheat pollen directly or indirectly.

The proteasomal degradation pathway is essential for many cellular processes, including the regulation of gene expression, the cell cycle, and responses to oxidative stress, and it also participates in plant pollen abortion. From the early stage of mononucleate pollen to the initial stage of trinucleate pollen, the expression level of ubiquitin-related modifier 1 gene was significantly higher in PMS wheat anthers than in fertile anthers, and the F-box protein gene was significantly up-regulated in PMS wheat anthers from the late stage of mononucleate pollen to the initial stage of binonucleate pollen. These genes are the promoters for pollen abortion of wheat PMS induced by the chemical hybridization agent SQ-1 [28].

Differential proteomic analysis of polyubiquitinated proteins associated with wheat male sterility identified 127 differentially expressed polyubiquitinated proteins, including heat shock protein 70, ATPase subunit, ubiquitin-related enzyme, glycosyltransferase, and 20S proteasome subunit. Proteins in PMS wheat anthers whose expression was up-regulated included those in the categories of protein metabolism, carbohydrate and energy metabolism, amino acid metabolism, and plant secondary metabolism. However, PMS wheat anthers expressed much lower levels of photosynthesis-related proteins. Alteration of polyubiquitinated proteins is associated with wheat male sterility [56].

**Fig 6:**
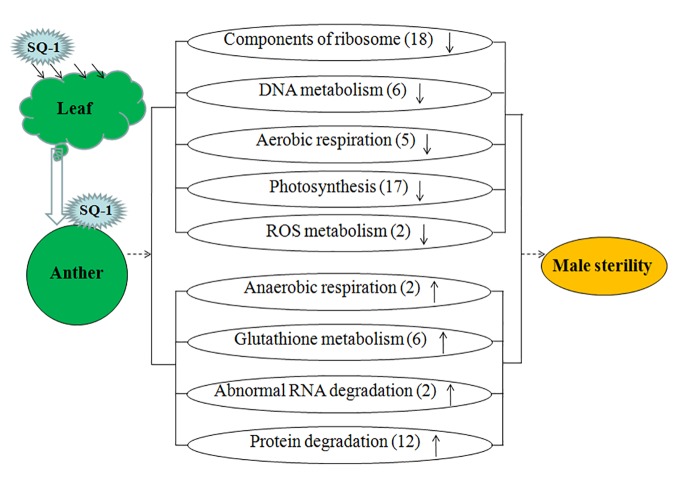
A simple mode of SQ-1–induced male sterility in wheat. Some important unigenes are related to wheat male sterility induced by SQ-1. “↑” in the ellipses indicates unigenes that were up-regulated in sterile wheat, “↓” in the ellipses indicates unigenes that were down-regulated, and “()” represents the number of differentially expressed unigenes.

Our results showed that most of the up-regulated unigenes were closely associated with the categories of protein degradation and glutathione metabolism. The down-regulated unigenes were related to the categories of “photosynthesis” (17 unigenes) and “ribosome” (18 unigenes) (Fig 6). The down-regulated expression of ribosomes affected biological protein synthesis, which was probably the key reason for wheat pollen abortion.

## Conclusions

This is the first study to obtain a large-scale SQ-1–induced male sterile wheat anther transcriptome dataset using high-throughput Illumina sequencing technology. Using functional annotation and enrichment analysis, we analyzed differentially abundant unigenes between fertile wheat and SQ-1–induced male sterile wheat and established a simple mode of SQ-1–induced male sterility in wheat (Fig 6).

After SQ-1 was sprayed on the leaves of wheat, it was transported from the leaves to the flowers. Its effects included down-regulated expression of numerous unigenes involved in “nucleotide metabolism,” “ribosome,” “aerobic respiration,” “photosynthesis,” and “ROS metabolism.” In addition, a large number of unigenes were up-regulated, including those that were closely associated with “anaerobic respiration,” “glutathione metabolism,” “abnormal RNA degradation,” and “protein degradation.” We identified a great number of important genes related to wheat male sterility and provided a framework for further mechanism studies on SQ-1–induced male sterility in wheat.
